# Prevalence and Factors Associated with Sarcopenia in Post-Menopausal Women with Rheumatoid Arthritis

**DOI:** 10.31138/mjr.260323.paf

**Published:** 2024-02-12

**Authors:** Dimitra Moschou, Michail Krikelis, Christos Georgakopoulos, Evangelia Mole, Efstathios Chronopoulos, Symeon Tournis, Clio Mavragani, Konstantinos Makris, Ismene Dontas, Susana Gazi

**Affiliations:** 1 Rheumatology Department, KAT General Hospital, Attica, Greece; 2 Laboratory for Research of the Musculoskeletal System “Theodoros Garofalidis”, School of Medicine, National and Kapodistrian University of Athens, Greece; 3 Department of Physiology, School of Medicine, National and Kapodistrian University of Athens, Greece; 4 Clinical Biochemistry Department, KAT General Hospital, Attica, Greece

**Keywords:** sarcopenia, IGF-I, rheumatoid arthritis, inflammation

## Abstract

**Objective/Aim:**

To estimate the prevalence of sarcopenia in post-menopausal women with rheumatoid arthritis (RA) and to investigate possible correlation with disease parameters.

**Methods:**

Eighty post-menopausal women with RA and thirty post-menopausal controls were enrolled in this cross-sectional study. RA patients were further divided in two groups according to the existence of sarcopenia. Sarcopenia was defined according to EWGSOP-II recommendations and osteoporosis as a T-score≤-2.5 in femoral neck bone mineral density. Biomarkers of bone turnover were determined. RA disease activity was calculated using the DAS28-ESR score and inflammatory markers (ESR, CRP). Functionality was calculated with the HAQ-DI score and seropositivity was determined according to RF and anti-CCP antibodies.

**Results:**

Thirty-two post-menopausal women with RA (39%) met the EWGSOP-II criteria for sarcopenia. None of the control subjects was detected with sarcopenia (p<0.0001). All parameters that define sarcopenia were significantly lower in the RA group. Sarcopenic RA patients had significantly lower mean BMI (27.1 kg/m2 vs. 30.5 kg/m2, p=0.008), daily physical activity (IPAQ score) (1213 vs 2867, p<0.0001), mean skeletal muscle mass (ASMI) (5.2 kg/m2 vs 6.6 kg/m2, p<0.0001) and handgrip strength (13.7 kg vs 20.1 kg, p<0.0001). No differences were observed in disease parameters or in biomarkers of bone turnover. IGF-1 was the only parameter that differed between the sarcopenic and non-sarcopenic RA patients (90.1 ng/ml vs 112.8 ng/ml, p=0.024).

**Conclusion:**

Sarcopenia is more common in RA patients. Sarcopenic RA patients had lower BMI, IPAQ, ASMI and handgrip strength. IGF-1 was the only parameter that was significantly lower in sarcopenic RA patients.

## INTRODUCTION

Rheumatoid arthritis (RA) is a systemic inflammatory autoimmune disease. It manifests as a mild oligoarticular syndrome or as a chronic progressive polyarthritis with impairment of functionality.^1^ Patients with RA are at greater risk for sarcopenia, osteoporosis, and associated morbidity compared to the general population.^2^

The first attempt to define sarcopenia is attributed to Irwin Rosenberg, who described it in 1989 as “a decline in muscle mass”. He used the Greek words “sarx” or “sarka,” which mean flesh, and “penia,” which means scarcity.^3^ The European Working Group on Sarcopenia in the Elderly (EWGSOP) in 2010 defined sarcopenia as “a syndrome characterised by progressive and generalised loss of skeletal muscle mass and strength with a risk of adverse outcomes, such as poor physical quality of life and death”.^4^ In 2019 the EWGSOP updated the definition of sarcopenia. The main differences are: 1) diagnosis requires documentation of low muscle strength and low muscle mass, while physical performance is used to categorise the severity of sarcopenia; and 2) new cut-off points were recommended.^5^ Although the term “sarcopenia” was originally intended to refer to the elderly, the increased prevalence of autoimmune diseases in younger patients has led to a special focus in this population. “Primary sarcopenia” refers to age-related sarcopenia without a recognised cause, whereas “secondary sarcopenia” happens when one or more causes, like RA, have been identified.^5^

The EWGSOP has established guidelines for diagnosing sarcopenia in clinical practice with the help of imaging modalities. The DXA method is broadly used for measuring muscle mass since it is a cheap, quantitative method, usually available in clinical practice, which shows a good correlation with more accurate measurements made through computed tomography and magnetic resonance tomography.^6^ Muscle strength is commonly assessed by using a handgrip dynamometer, whereas physical performance can be measured by use of the Short Physical Performance Battery (SPPB).^7^

Trying to designate the additional morbidity caused by a combination of osteoporosis and sarcopenia, Duque was the first investigator to use the term “osteosarcopenia”. According to this definition, osteosarcopenia is a syndrome defined by a combination of low bone mineral density (osteopenia/osteoporosis) and decreased muscle mass, strength, and/or functional capacity (sarcopenia) resulting in a greater number of falls, fractures, and hospitalisation.^8^

It has been documented that inflammation is an important stimulus for both sarcopenia and osteosarcopenia in RA patients. The sarcopenia observed in RA is due to an overproduction of proinflammatory cytokines (IL-6, TNF-α and IL-1β), oxidative stress and reduced physical activity, which in combination lead to exaggerated catabolism of proteins and subsequent muscular breakdown.^9^ Furthermore, failure to control well-established active disease (a common clinical scenario) leads to prolonged iatrogenic exposure to glucocorticoids, which adds to the loss of muscle mass.^10^ For this reason, treatment with targeted disease-modifying drugs drastically prevents protein catabolism caused by inflammatory cytokines.^11^

Despite the above evidence, the relationship between sarcopenia and inflammatory arthritis remains under investigation. The aim of this cross-sectional study is to estimate the prevalence of sarcopenia in a population of post-menopausal women with RA and to investigate a possible correlation with clinical and biochemical parameters.

## MATERIALS AND METHODS

### Study design

This is a cross-sectional case-control study including post-menopausal women with rheumatoid arthritis and healthy controls.

The post-menopausal status was intentionally selected to tackle the influence of sex hormones in the development of the musculoskeletal system.^7^

RA diagnosis was made according to 2010 ACR/EULAR classification criteria.^12^ Exclusion criteria were: pre-menopausal status, difficulty to walk by oneself, joint arthroplasty with metallic implants, major hand and/or foot joint deformities, patients with malignancy and patients with chronic comorbidities (renal failure, liver failure).

By applying a convenience sampling method, we randomly recruited subjects who visited the ambulatory outpatients of the Rheumatology Department of “KAT” General Hospital, Athens, Greece. Between January 2019 and February 2020. Patients with already diagnosed RA under treatment and healthy controls were recruited at a random ratio.

The institutional scientific and ethics committee approved the study protocol (decision number: 8690/8-7-2019). All participants signed an informed consent form.

### Study measures

Sarcopenia diagnosis was made upon documentation of low muscle strength and low muscle mass. Physical performance was used to further categorise the severity of sarcopenia based on the updated EWGSOP2 sarcopenia definition criteria.^13^ All the above parameters were evaluated by a trained occupational therapist. Body muscle mass measurements were made by whole-body dual-energy X-ray absorptiometry (DXA Lunar Prodigy Pro, GE). The Appendicular Skeletal Muscle Index (ASMI) was calculated as the sum of upper and lower limb muscle mass divided by squared height (kg/m^2^). According to the ESPEN (European Society of Clinical Nutrition and Metabolism) Diagnostic Criteria for Sarcopenia the cut-off values for the ASMI index in women were set at 5.45 kg/m^2^.^14^ Subjects with values lower than 5.45 kg/m^2^ were characterised as having “low muscle mass”.

Bone mineral density (BMD) at the lumbar spine and femoral neck was also calculated by DXA. Osteoporosis was defined as low bone mineral density (BMD) with a T-score ≤ −2.5.^15^

Physical performance was assessed with the Short Physical Performance Battery (SPPB) tool.^16^ This tool consists of 3 tests and the score ranges from 0 to 12 points, where ≥10 points indicate good functioning, 7–9 points indicate moderate functioning and ≤6 points indicate reduced functioning.^17^ Moreover, patients and controls completed the International Physical Activity Questionnaire (IPAQ), a standardised questionnaire used to evaluate their daily physical activity.^18^

The assessment of muscle strength was performed with use of the hand dynamometer method (JAMAR digital hand dynamometer: Patterson Medical, IL, USA). Cut-off levels were defined as <20 kg for women, according to the EWGSOP sarcopenia diagnostic criteria also endorsed by ESPEN.^19^

Disease specific data included: the date of diagnosis, the duration of the disease, the seropositivity for anti-cyclic citrullinated peptide (anti-CCP) and rheumatoid factor (RF) and the treatments administered for both RA and osteoporosis. Disease activity was estimated with use of the DAS28-ESR score. ^20^ Patient functionality was assessed with use of the Health Assessment Questionnaire Disability Index (HAQ-DI).^21^

### Biochemical measurements

All blood samples were collected after overnight fasting, centrifuged within 1h from the collection (at 3000 rpm for 10 min), aliquoted and stored at −80 °C until tested. Serum total calcium magnesium and phosphorus were measured with a colorimetric assay on Architect-8000 Automated Clinical Chemistry analyser (Abbott, Chicago, IL). The total analytical imprecision of both assays in our laboratory is <1.0%. Serum creatinine measurements were performed with a Jaffe modified method traceable to IDMS on Architect-8000 Automated Clinical Chemistry analyser (Abbott, Chicago, IL). The total analytical imprecision of this assay in our laboratory is <2.5%. Serum total alkaline phosphatase activity was measured with a colorimetric method that is traceable to IFCC reference measurement procedure on Architect-8000 Automated Clinical Chemistry analyser (Abbott, Chicago, IL).

Serum albumin was measured with a colorimetric BCG (bromocresol green) assay. On Architect-8000 Automated Clinical Chemistry analyser (Abbott, Chicago, IL) The total analytical imprecision of this assay in our laboratory is <1.6%. Serum levels of total 25-hydroxy-vitamin D [25(OH) D3] electrochemiluminescence immunoassay (ECLIA) on Cobas e411 (Roche Diagnostics GmbH, Mannheim, Germany). Serum levels of PTH were measured with a second-generation PTH assay on the same analyser. The total analytical imprecision of these assays in our laboratory is <4.7% and <4.0% respectively. Total pro-collagen type I N-terminal propeptide (total-PINP), C-terminal telopeptide of collagen I (CTX), and N-MID-osteocalcin (OC) were measured by electrochemiluminescence immunoassays on Cobas e411 automated analyser (Roche Diagnostics GmbH, Mannheim, Germany) according to the manufacturer’s instructions. The total analytical imprecision of these assays in our laboratory were respectively: <4.5% <3.5%, and <3.5% respectively. Ιnsulin-like growthfactor-1 (IGF-1) was measured by an electrochemiluminescence immunoassays on Cobas e411 automated analyser (Roche Diagnostics GmbH, Mann-heim, Germany) according to the manufacturer’s instructions. The total analytical imprecision of this assay in our laboratory was <4.0%.

### Statistical analysis

This is a case-control study and thus comparisons were performed between RA patients and healthy controls. Further analysis was performed within the RA population itself, where comparisons were made between subjects with sarcopenia and those without.

Continuous variables were tested for normality with the Shapiro-Wilk test and followed a normal distribution (>0.05). Continuous variables are expressed in mean values and standard deviations (mean value ± standard deviation). Differences between mean values were evaluated with the student’s t-test. Categorical values are expressed as totals (n) and percentages (%). Differences between categorical variables were evaluated with the chi-square (x2) test. Where appropriate, associations are expressed as odds ratios (OR) and 95% confidence intervals (95% CI).

A multivariate linear regression analysis was used to investigate the calculated variables as possible predictors of sarcopenia in the RA population. The model’s R-squared value (R2), beta coefficients and the relative statistical significance were recorded. The receiver operating characteristic (ROC) curve was used to assess the overall diagnostic performance of IGF-I to predict sarcopenia in the RA population. The area under the curve (AUC) and statistical significance were calculated.

Statistical significance was set at p<0.05.

Statistical analysis was performed by use of a licensed product of the IBM SPSS Software v.24.

## RESULTS

Based on our convenience sampling method, 80 post-menopausal women with RA and 30 healthy controls were recruited. Baseline demographics and study variables are presented in **Table 1**.

**Table 1.** Study variables and comparisons between patients with RA and controls.

	**All patients (n=80)**	**Controls (n=30)**	**P-value**

**Demographics**			

Age (years)	66 (±9)	64 (±7)	0.08
Mean (±SD)			

BMI (kg/m2)	29.2 (±5.5)	26.3 (±4.1)	0.012
Mean (±SD)			

**Disease parameters**			

Anti-CCP n (%)	31 (40.8%)	NA	

RF n (%)	31 (41.3%)	NA	

Glucocorticoids n (%)	37 (47.4%)	NA	-

MTX n (%)	40 (51.3%)	NA	-

LEF n (%)	18 (23.1%)	NA	-

HCQ n (%)	3 (3.8%)	NA	-

bDMARD n (%)	46 (59%)	NA	-

ESR (mmHg)	26 (±18)	15 (±7)	0.001
Mean (±SD)			

CRP (mg/dl)	1.23 (±1.92)	0.34 (±0.13)	0.012
Mean (±SD)			

DAS 28-ESR	3.89 (±1.70)		
Mean (±SD)	21 (26.9%)		
High disease activity n (%)	27 (34.6%)	NA	-
Moderate disease activity n (%)	13 (16.7%)		
Low disease activity n (%)	17 (21.8%)		
Remission n (%)			

HAQ-DI	0.89 (±0.58)		
Mean (±SD)	41 (52.6%)		
Functional remission n (%)	32 (41.0%)	NA	-
Moderate disability n (%)	5 (6.4%)		
Severe disability n (%)			

**Osteoporosis parameters**			

Osteoporosis n (%)	22 (28.2%)	11 (37.9%)	0.260

Bisphosphonates n (%)	32 (41%)	5 (16.7%)	0.050

Denosumab n (%)	6 (7.7%)	1 (3.3%)	0.407

Teriparatide n (%)	0	0	-

Calcium supplement n (%)	46 (59%)	5 (16.7%)	0.007

Vitamin D supplement n (%)	51 (65.4%)	8 (26.7%)	0.030

Fracture n (%)	30 (38.5%)	9 (30%)	0.931
Hip fracture n (%)	2 (2.6%)	2 (6.7%)	0.323
Vertebral fracture n (%)	4 (5.1%)	3 (10%)	0.189
Other fracture n (%)	25 (32.1%)	5 (16.7%)	0.257

**Sarcopenia parameters**			

Sarcopenia n (%)	32 (39%)	0	<0.0001

ASMI (kg/m2)	6.05 (±0.86)	6.72 (±1.61)	0.006
Mean (±SD)			

Hand grip (kg)	17.5 (±12.5)	31.1 (±10.4)	<0,0001
Mean (±SD)			

SPPB	7 (±2)	11 (±1)	<0.0001

IPAQ	2186 (±2062)	2438 (±1452)	0.696
Mean (±SD)	19 (23.8%)	2 (6%)	
Low <600 n (%)			
Moderate 600–3000 n (%)	36 (45%)	19 (63.3%)	
High >3000 n (%)	23 (29%)	9 (30%)	

**Osteosarcopenia**			

Osteosarcopenia n (%)	12 (15.4%)	0	0.009

**Bone metabolism**			

Ca (mg/dL)	9.4 (±0.5)	9.2 (±0.3)	0.039
Mean (±SD)			

Alb (gr/dL)	4.2 (±0.3)	4.3 (±0.8)	0.497
Mean (±SD)			

P (mg/dL)	3.5 (±0.5)	3.6 (±0.4)	0.161
Mean (±SD)			

Mg (mg/dL)	1.9 (±0.4)	2.4 (±0.5)	<0.0001
Mean (±SD)			

Cr (mg/dL)	0.76 (±0.16)	0.7 (±0.08)	0.146
Mean (±SD)			

ALP (IU/L)	68 (±24)	65 (±17)	0.470
Mean (±SD)			

BALP (ng/mL)	12.7 (±4.6)	19.6 (±10.6)	0.002
Mean (±SD)			

PTH (pg/mL)	53.9 (±22.3)	38.7 (±15.9)	0.002
Mean (±SD)			

25(OH)D3 (ng/mL)	30.1 (±10.1)	24.9 (±11.1)	0.021
Mean (±SD)			

TSH (μU/mL)	1.5 (±1.1)	2.2 (±2.9)	0.112
Mean (±SD)			

OCN (mg/L)	16.9 (±8.9)	19.7 (±7.1)	0.132
Mean (±SD)			

CTx (pg/mL)	0.28 (±0.18)	0.34 (±0.17)	0.102
Mean (±SD)			

IGF-1 (ng/mL)	103.4 (±42.9)	121.3 (±34.9)	0.053
Mean (±SD)			

Data are presented as means (± SD) or n (%). BMI: Body mass index; Anti-CCP: anti-cyclic citrullinated peptide antibody; RF: Rheumatoid factor; MTX: Methotrexate; LEF: Leflunomide; HCQ: Hydroxychloroquine; bDMARD, biological disease-modifying antirheumatic drugs; ESR: Erythrocyte Sedimentation Rate; CRP: C-reactive protein; DAS28-ESR: disease activity score in 28 joints-erythrocyte sedimentation rate; HAQ-DI: Health Assessment Questionnaire Disability Index; ASMI: skeletal muscle mass index; SPPB: Short Physical Performance Battery; IPAQ: International Physical Activity Questionnaire; Ca: calcium; Alb: albumin; P: Phosphorus; Mg: magnesium; Cr: creatinine; ALP: alkaline phosphatase; BALP: bone alkaline phosphatase; PTH: parathyroid hormone; 25(OH)D3: 25-hydroxyvitamin D3; TSH: thyroid stimulating hormone; OCN: osteocalcin; CTx: cross-linked C-telopeptide of type I collagen; IGF-1: insulin-like growth factor 1; NA: not applicable.

Patients’ mean age and BMI were 66± 9 years and 29.2±5.5 kg/m2 respectively. There was a statistically significant difference in BMI values between patients and controls, reflecting the well-known prevalence of increased body weight in RA patients.^22^ About one third (n=31, 41%) of the RA patients had seropositive disease. All patients were administered disease modifying treatments, 47.4% were receiving low dose glucocorticoids (< 7.5 mg prednisolone daily) and 59% were receiving biological therapies (bDMARDs). The mean DAS28-ESR index was 3.89±1.70. In the RA group, 21 patients (26.9%) had high disease activity, 27 (34.6%) moderate, 13 (16.7%) low and 17 (21.8%) were in remission. The mean HAQ-DI was 0.89±0.58. Half the patients (52.6%) had achieved functional remission (HAQ-DI ≤ 0.5) when they entered the study.

Mean ASMI was 6.05±0.86 kg/m^2^ and 37.5% of the patients satisfied the criteria for “low muscle mass” (ASMI< 5.45 kg/m^2^). Mean grip strength was 17.5±12.5 kg and about 67.5% were below the cut-off value for low grip strength <20 kg. A third of the patients (27.5%) were below the cut-off value for low physical performance according to the SPPB battery. In total, 32 (39%) post-menopausal women with RA met the EWGSOP criteria for sarcopenia. No sarcopenia was observed in the control group, whereas all the parameters used to define sarcopenia were significantly lower in the patient group. As such, the OR for sarcopenia in our RA population was calculated at 19.1 (95% CI: 2.5 – 147.8).

There were no differences between patients and controls regarding daily physical activity as calculated with the IPAQ score (p=0.696).

The prevalence of osteoporosis in the RA group was 28.2% (n=22) vs 37.9% (n=11) in controls (p=0.260). There was no significant difference between patients and controls in terms of fragility fractures (p=0.931) or administered anti-osteoporotic treatment regimens. RA patients tended to receive calcium and cholecalciferol supplementation at an increased rate, reflecting the adherence to therapeutic guidelines for corticosteroid-induced osteoporosis in inflammatory diseases.^23^ There were no remarkable differences in bone metabolism parameters between patients and controls.

Last, 12 patients (15.4%) and no controls were diagnosed with osteosarcopenia (p=0.09).

**Table 2** summarises the comparisons between RA patients with and without sarcopenia. Sarcopenic patients had a lower BMI (27.1 kg/m^2^ vs 30.5 kg/m2, p=0.008) and decreased daily physical activity (IPAQ) (1213 vs 2867, p<0.0001). They also had a lower skeletal muscle mass (5.2 kg/m^2^ vs 6.6 kg/m^2^, p<0.0001) and lower scores in handgrip strength (13.7 kg vs 20.1 kg, p<0.0001). Interestingly, no differences were reported in the disease parameters or in parameters associated with osteoporosis.

**Table 2.** Comparisons between RA patients with and without sarcopenia.

	**Patients with sarcopenia (n=32, 39%)**	**Patients without sarcopenia (n=48, 61%)**	**P-value**

	**Demographics**		

Age (years)	67 (±9)	65 (±9)	0.150
Mean (±SD)			

BMI (kg/m2)	27.1 (±5.9)	30.5 (±4.9)	0.008
Mean (±SD)			

	**Disease parameters**		

Anti-CCP n (%)	13 (41.9%)	18 (40%)	0.866

RF n (%)	12 (40%)	19 (42.2%)	0.848

Glucocorticoids n (%)	14 (45.2%)	23 (48.9%)	0.744

MTX n (%)	16 (51.1%)	24 (51.6%)	0.962

LEF n (%)	11 (35.5%)	7 (14.9%)	0.035

HCQ n (%)	1 (3.2%)	2 (4.3%)	1.000

bDMARD n (%)	16 (51.6%)	30 (63.8%)	0.283

ESR (mmHg)	30 (±22)	24 (±14)	0.196
Mean (±SD)			

CRP (mg/dl)	1.29 (±2.25)	1.19 (±1.69)	0.823
Mean (±SD)			

DAS 28-ESR	3.75 (±1.49)	3.99 (±1.83)	0.525
Mean (±SD)			
High disease activity n (%)	5 (16.1%)	16 (34.0%)	
Moderate disease activity n (%)	15 (48.4%)	12 (25.5%)	
Low disease activity n (%)	5 (16.1%)	8 (17.0%)	
Remission n (%)	6 (19.4%)	11 (23.4%)	

HAQ-DI	0.78±0.53	0.97±0.60	0.148
Mean (±SD)			
Functional remission n (%)	19 (61.3%)	22 (46.8%)	
Moderate disability n (%)	10 (32.3%)	22 (46.8%)	
Severe disability n (%)	2 (6.5%)	3 (6.4%)	

**Osteoporosis parameters**			

Osteoporosis n (%)	12 (38.7%)	10 (21.3%)	0.094

Bisphosphonates n (%)	15 (48.4%)	17 (36.2%)	0.283

Denosumab n (%)	3 (9.7%)	3 (6.4%)	0.677

Teriparatide n (%)	0	0	-

Calcium supplement n (%)	21 (67.7%)	25 (53.2%)	0.201

Vitamin D supplement n (%)	22 (71%)	29 (61.7%)	0.400

Fracture n (%)	11 (35.5%)	19 (40.4%)	0.661
Hip fracture n (%)	0	2 (4.3%)	0.515
Vertebral fracture n (%)	0	4 (8.5%)	0.147
Other fracture n (%)	11 (35.5%)	14 (29.8%)	0.598

**Sarcopenia parameters**			

ASMI (kg/m2)	5.20 (±0.35)	6.61 (±0.58)	<0.0001
Mean (±SD)			

Hand grip (kg)	13.7 (±8.6)	20.1 (±14.0)	0.014
Mean (±SD)			

SPPB	7 (±2)	7 (±3)	0.318
Mean (±SD)			

IPAQ	1213 (±1073)	2867 (±2297)	
Mean (±SD)			
<600 n (%)	13 (68.4%)	6 (31.6%)	<0.0001
600-3000 n (%)	16 (44%)	20 (56%)	
>3000 n (%)	2 (8.6%)	21 (91.3%)	

**Bone metabolism**			

Ca (mg/dL)	9.2 (±1.4)	9.5 (±0.5)	0.177
Mean (±SD)			

Alb (gr/dL)	4.2 (±0.4)	4.3 (±0.3)	0.106
Mean (±SD)			

P (mg/dL)	3.5 (±0.6)	3.4 (±0.5)	0.372
Mean (±SD)			

Mg (mg/dL)	1.9 (±0.2)	1.9 (±0.5)	0.426
Mean (±SD)			

Cr (mg/dL)	0.79 (±0.19)	0.75 (±0.13)	0.222
Mean (±SD)			

ALP (IU/L)	74 (±31)	64 (±17)	0.058
Mean (±SD)			

BALP (ng/mL)	13.6 (±5.8)	11.9 (±3.3)	0.156
Mean (±SD)			

PTH (pg/mL)	57.1 (±23.3)	51.9 (±21.6)	0.319
Mean (±SD)			

25(OH)D3 (ng/mL)	30.8 (±12.2)	29.6 (±8.5)	0.631
Mean (±SD)			

TSH (μU/mL)	1.3 (±0.9)	1.7 (±1.2)	0.202
Mean (±SD)			

OCN (mg/L)	18.7 (±9.9)	15.6 (±8.1)	0.167
Mean (±SD)			

CTx (pg/mL)	0.32 (±0.21)	0.25 (±0.15)	0.099
Mean (±SD)			

IGF-1 (ng/mL)	90.1 (±37.2)	112.8 (±44.6)	0.024
Mean (±SD)			

Data are presented as means (± SD) or n (%). BMI: Body mass index; Anti-CCP: anti-cyclic citrullinated peptide antibody; RF: Rheumatoid factor; MTX: Methotrexate; LEF: Leflunomide; HCQ: Hydroxychloroquine; bDMARD: biological disease-modifying antirheumatic drugs; ESR: Erythrocyte Sedimentation Rate; CRP: C-reactive protein; DAS28-ESR: disease activity score in 28 joints-erythrocyte sedimentation rate; HAQ-DI: Health Assessment Questionnaire Disability Index; ASMI: skeletal muscle mass index; SPPB: Short Physical Performance Battery; IPAQ: International Physical Activity Questionnaire; Ca: calcium; Alb, albumin; P: Phosphorus; Mg: magnesium; Cr: creatinine; ALP: alkaline phosphatase; BALP: bone alkaline phosphatase; PTH: parathyroid hormone; 25(OH)D3, 25-hydroxyvitamin D3; TSH: thyroid stimulating hormone; OCN: osteocalcin; CTx: cross-linked C-telopeptide of type I collagen; IGF-1: insulin-like growth factor 1; NA: not applicable.

Multivariate linear regression analysis indicated IGF-I (p=0.029) and IPAQ score (p<0.001) as independent factors to predict sarcopenia in RA patients (R2 79%, model significance of < 0.0001). Disease parameters, factors associated with osteoporosis or bone metabolism markers did not contribute significantly to sarcopenia in our model.

**Figure 1.**
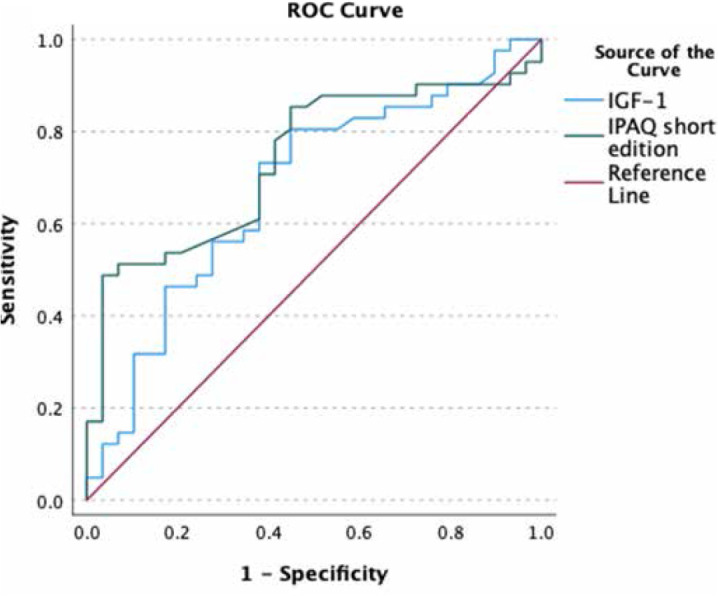
ROC curve for the relationship between IGF-1, IPAQ, and sarcopenia in the RA population.

ROC analysis revealed IGF-1 as a possible predictor of sarcopenia in the RA (AUC=0.675, 95% CI: 0.545–0.804), p=0.008 and an overall model quality of 55%. Similar results apply to IPAQ regarding the prediction of sarcopenia in our RA population (AUC=0.737, 95% CI: 0.620–0.855), p<0.0001 and an overall model quality of 62% **(Figure 1)**.

## DISCUSSION

To our knowledge, this is one of the few studies that provide evidence about the increased prevalence of sarcopenia in patients with rheumatoid arthritis. According to the literature, sarcopenia disproportionately affects such patients with a reported prevalence of 17 to 60%, whereas an aggressive disease profile (e.g., seropositive disease), disease activity for prolonged periods and treatment with corticosteroids result to reduced muscle mass and frailty that escalate to sarcopenia.^24^ Despite the application of exclusion criteria, ours is a real-world RA population and the calculated prevalence of sarcopenia is 39%. Our findings suggest that post-menopausal women with RA have a 19-times increased risk to suffer from sarcopenia. According to the revised EWGSOP-II criteria, sarcopenic patients characterised by low muscle strength, low muscle quantity/quality and low physical performance suffer from “severe sarcopenia”.^25^ By using a random sampling method, we indicated that almost half of our RA patients had significantly lower scores in all three. This finding possibly suggests that, when associated with rheumatoid arthritis, sarcopenia is usually severe.

The low handgrip strength observed in our study is a rather expected outcome given that RA commonly affects the hand joints and leads to debilitating deformities.^26^ However, within the RA group itself, sarcopenic RA patients had significantly lower handgrip strength scores when compared to non-sarcopenic ones. This finding helps us conclude that muscle wasting worsens the already diminished functionality of the hands in RA patients.

Regarding the domain of physical activity, our study indicates that post-menopausal women with rheumatoid arthritis score low in physical performance (as measured with the SPPB test) and at the same time they lead a sedentary way of life (as measured with the IPAQ questionnaire). While the first parameter is included in the definition of sarcopenia per se, the second one seemed to associate well with prevailing sarcopenia in our RA population. In parallel, the HAQ-DI index, commonly used to record the functionality of RA patients, did not correlate with sarcopenia in our study. We suggest that the IPAQ questionnaire might be of high utility in clinical practice, with low scores raising a suspicion of subclinical muscle wasting in RA patients, even though they record normal HAQ-DI scores. Thus, recording the baseline daily physical activity of RA patients will prove useful in designing interventions that effectively improve their physical performance in the long term.^27^

An important finding of our study are the low values of IGF-1 calculated in the sera of RA patients with sarcopenia. IGF-1 has already been recognised as a key molecule in the pathogenesis of sarcopenia in post-menopausal women: reduced serum concentrations of IGF-I correlate well with sarcopenia in geriatric patients, assuming an anabolic mechanism where IGF-I promotes myoblast proliferation, differentiation and stimulation of satellite cell proliferation and muscle protein synthesis.^28^ Moreover, experiments in animal models indicate that the systemic action of pro-inflammatory cytokines leads mainly to hepatic GH resistance and suppression of IGF-1 action in target tissues. This is performed through the downregulation of growth hormone receptor (GH), the upregulation of the members of the SOCS family that negate the action of GH and an increased clearance of IGF-I through catabolism of IGF-I bindings proteins (IGFBPs).^29^ Our study contributes to this literature and assumes a possible role of IGF-I in the pathogenesis of RA sarcopenia. Low serum values of this molecule could serve as a screening test for post-menopausal women with RA prone to develop sarcopenia. Thus, in parallel to reducing disease activity with targeted disease modifying drugs, achieving the ideal IGF-I serum concentrations through dietary interventions in cooperation with clinical nutritionists can benefit RA patients.^30, 31^

Last, in our study, 28% of RA patients suffered from osteoporosis and were administered anti-osteoporotic treatments. Considering the frequent co-existence of sarcopenia and osteoporosis in inflammatory arthritis, we were able to recognise a sub-population of osteosarcopenic RA patients (15%). These patients run the highest risk for muscle wasting, falls and subsequent osteoporotic fractures, require a tighter monitoring for disease control and should be timely considered as candidates for a more aggressive anti-osteoporotic treatment scheme.^32^

We believe that our study paves the way towards investigating sarcopenia in patients with rheumatoid arthritis. By suggesting IGF-I as a molecule that bridges inflammation and muscle wasting and by recognising decreased physical performance as a crucial contributor to sarcopenia, we provide clinicians with additional tools to measure and combat sarcopenia in this population. However, the cross-sectional design of the study and the convenience sampling method take a considerable impact in the generalisation of our results. Still, the provision of real-world evidence and the utilisation of accurate laboratory measurements/imaging modalities do increase the reliability of our findings and call for further research into the matter.

In conclusion, it is important to note that disease activity, patients’ weight, daily physical activity, muscle mass and bone quality are all modifiable factors that should be kept in mind when treating RA patients. Possible lifestyle interventions, exercise schedules and suitable management of osteoporosis could accompany disease modifying drugs to prevent sarcopenia and help our patients achieve a better quality of life.
